# Impact of biomarker use recommendations on clinical practice in Italian Memory Clinics

**DOI:** 10.1002/alz70860_105603

**Published:** 2025-12-23

**Authors:** Cristina Festari, Claudio Singh Solorzano, Stefania Orini, Eleonora Castagna, Matteo Cotta Ramusino, Fabrizia D'Antonio, Adolfo Di Crosta, Alessia Masòtino, FEDERICO MASSA, Alice Mazzonetto, Massimiliano Panigutti, Noemi Ravì, Michela Pievani, Laura Bonanni, Giuseppe Bruno, Annachiara Cagnin, Roberto Gatta, Giovanni B. Frisoni

**Affiliations:** ^1^ IRCCS Istituto Centro San Giovanni Di Dio Fatebenefratelli, Brescia, Italy; ^2^ Università degli Studi di Brescia, Brescia, Italy; ^3^ Sapienza University of Rome, Roma, Italy; ^4^ IRCCS Mondino Foundation, Pavia, Italy; ^5^ Sapienza University of Rome, Roma, Lazio, Italy; ^6^ University G. d'Annunzio of Chieti‐Pescara, Chieti, Italy; ^7^ University of Genova, Genova, Italy; ^8^ IRCCS Ospedale Policlinico San Martino, Genova, Italy; ^9^ University of Padova, Padova, Italy; ^10^ Sapienza University of Rome, Rome, Italy; ^11^ Geneva University Hospitals, Geneva, Switzerland; ^12^ University of Geneva, Geneva, Switzerland

## Abstract

**Background:**

Assessing the feasibility and implementation of recommendations in routine clinical practice is uncommon. This study aimed to evaluate the adherence of three memory clinics to the biomarker‐based diagnostic approach outlined in the Italian Inter‐societal Consensus Recommendations (IICR; Boccardi, 2020).

**Method:**

We retrospectively reviewed the medical records of consecutive new patients with cognitive complaints from two periods: 2018‐19 (T0) and 2022‐23 (T1), before and after the publication of the IICR. Adherence was measured using a modified Adherence Index (AI) that included five domains: clinical visit, blood exam, neuropsychological assessment, structural neuroimaging, and biomarkers. The clinical value of each domain, except for biomarkers, was weighted according to the expert opinions of three neurologists. For biomarkers, adherence to IICR was assessed based on the initial diagnostic hypothesis. AI scores for each domain ranged from 0 to 1, with the global AI ranging from 0 to 5, where higher scores indicated better adherence. Statistical comparisons were performed using the Mann‐Whitney U Test and the chi‐squared test (Χ2).

**Result:**

Of the 1,035 medical charts reviewed, 317 involved a biomarker‐based diagnostic work‐up (T0=178; T1=139). Following the publication of the recommendations, the global AI significantly improved from 2.09±1.04 to 2.28±1.04 points (U=8107; *p* = 0.047). In T1, the diagnostic process showed a more thorough neuropsychological assessment (U=9492; *p* <0.001) and more appropriate use of biomarkers (X2 (3)=39.66; *p* <0.001).

**Conclusion:**

The publication of the IICR had a positive impact on the clinical practice of Italian memory clinics, enhancing the recognition of the importance of comprehensive neuropsychological assessments and a more standardized use of biomarkers.

